# Higher Global Diet Quality Score Is Associated with Less 4-Year Weight Gain in US Women

**DOI:** 10.1093/jn/nxab170

**Published:** 2021-10-23

**Authors:** Teresa T Fung, Yanping Li, Sabri Bromage, Shilpa N Bhupathiraju, Carolina Batis, Wafaie Fawzi, Michelle D Holmes, Meir Stampfer, Frank B Hu, Megan Deitchler, Walter C Willett

**Affiliations:** Department of Nutrition, Simmons University, Boston, MA, USA; Department of Nutrition, Harvard TH Chan School of Public Health, Boston, MA, USA; Department of Nutrition, Harvard TH Chan School of Public Health, Boston, MA, USA; Department of Nutrition, Harvard TH Chan School of Public Health, Boston, MA, USA; Department of Nutrition, Harvard TH Chan School of Public Health, Boston, MA, USA; Channing Division of Network Medicine, Department of Medicine, Brigham and Women's Hospital, Harvard Medical School, Boston, MA, USA; CONACYT—Health and Nutrition Research Center, National Institute of Public Health, Cuernavaca, Mexico; Department of Global Health and Population, Harvard TH Chan School of Public Health, Boston, MA, USA; Channing Division of Network Medicine, Department of Medicine, Brigham and Women's Hospital, Harvard Medical School, Boston, MA, USA; Department of Epidemiology, Harvard TH Chan School of Public Health, Boston, MA, USA; Channing Division of Network Medicine, Department of Medicine, Brigham and Women's Hospital, Harvard Medical School, Boston, MA, USA; Department of Epidemiology, Harvard TH Chan School of Public Health, Boston, MA, USA; Department of Nutrition, Harvard TH Chan School of Public Health, Boston, MA, USA; Channing Division of Network Medicine, Department of Medicine, Brigham and Women's Hospital, Harvard Medical School, Boston, MA, USA; Intake—Center for Dietary Assessment, FHI Solutions, Washington, DC, USA; Department of Nutrition, Harvard TH Chan School of Public Health, Boston, MA, USA; Channing Division of Network Medicine, Department of Medicine, Brigham and Women's Hospital, Harvard Medical School, Boston, MA, USA

**Keywords:** diet quality, weight, women, obesity, epidemiology

## Abstract

**Background:**

We have developed a simple and globally applicable tool, the Global Diet Quality Score (GDQS), to measure diet quality.

**Objectives:**

To test the utility of the GDQS, we examined the associations of the GDQS with weight change and risk of obesity in US women.

**Methods:**

Health, lifestyle, and diet information were collected from women (*n* = 68,336) in the Nurses’ Health Study II (aged 27–44 y in 1991) through repeated questionnaires (1991–2015). The GDQS has 25 food groups (maximum = 49 points) and scoring higher points reflects a healthier diet. The association between GDQS change in 4-y intervals and concurrent weight change was computed with linear models adjusted for confounders.

**Results:**

Mean ± SD weight gain across 4-y periods was 1.68 ± 6.26 kg. A >5-point improvement in GDQS was associated with −1.13 kg (95% CI: −1.19, −0.77 kg) weight gain compared with a score change of <±2 points. For each 5-point increase, weight gain was 0.83 kg less for age <50 y compared with 0.71 kg less for age ≥50 y (*P*-interaction < 0.05). A >5-point score decrease was associated with 1.13 kg (95% CI: 1.04, 1.22 kg) more weight gain in women aged <50 y and 0.81 kg more (95% CI: 0.63, 0.98 kg) in women aged ≥50 y. Compared with little change in score, obesity RR was 0.77 (95% CI: 0.74, 0.81) for a >5-point increase and 1.32 (95% CI: 1.26, 1.37) for a >5-point decrease. Risk of obesity did not differ by age. Compared with other diet quality scores, the Alternate Healthy Eating Index-2010 had somewhat stronger associations than the GDQS (*P* < 0.05) but the GDQS had stronger associations than the Minimum Dietary Diversity for Women score (*P* < 0.05).

**Conclusions:**

Improvement of diet quality as measured by the GDQS was associated with less weight gain and risk of obesity in US women. The association was stronger for women aged <50 y. Associations similar in direction and magnitude were observed between the GDQS and obesity across age groups.

## Introduction

Obesity is a global health problem. In 2017 the mortality attributed to high BMI was estimated to be 2.4 million (and 77 million disability-adjusted life years) globally (1). Overweight and obesity in early adulthood has also been associated with higher risks of obesity-related cancer (2), diabetes (3), and cardiovascular mortality (4, 5). Weight gain in adulthood ≥5 kg has also been associated with higher risks of all-cause mortality (6), cardiovascular disease (7), and obesity-related cancers (8). Countries with the highest obesity-related mortality rates span across the range of economic development levels with North America, North Africa, the Middle East, Eastern Europe, and Central Asia at the top (1).

In observational studies, better diet quality (9) or improvement of diet quality was associated with less weight gain (10), especially in younger adults (11). In addition, improvement of diet quality was also shown to prevent weight gain in a randomized trial among reproductive-aged women (12). Healthy diets tended to be higher in fiber and proportionally higher in minimally processed carbohydrates than in refined carbohydrates. Combined, these 2 characteristics may limit the surge in glycemic response (13) and, hence, are less likely to stimulate hunger (14). Consequently, a healthy diet may prevent overeating.

In order to monitor diet quality globally in a consistent manner, a universal metric is a necessity. The need for a rapid dietary screening tool for clinical settings has also been raised by the American Heart Association (15). To be suitable for use in regions of different economic development stages, this metric needs to be sensitive enough to both reflect nutrient adequacy and predict common chronic disease risk. We have developed the Global Diet Quality Score (GDQS) based on the Prime Diet Quality Score (16). The GDQS is associated with nutrient adequacy and lower odds of a low hemoglobin concentration in low- and middle-income countries (17) (see elsewhere in this Supplement). As part of evaluating the utility of the GDQS in high-income countries, we assessed its associations with weight gain and obesity in a cohort of US women. In this analysis, we specifically examined concurrent changes in the GDQS and body weight because weight change can occur rapidly in response to changes in diet. Moreover, the prospective approach that uses past diet to predict future weight change cannot capture the relevant time frame effectively. As a result, we examined the relation between 4-y change in GDQS and concurrent weight change, as well as risk of obesity, in women of reproductive age and older.

## Methods

The Nurses’ Health Study II (NHS II) is an ongoing prospective cohort study that began in 1989 with 116,430 US female Registered Nurses aged 25–42 y (18). Every 2 y, the nurses provided lifestyle, health, and medication information through a self-reported questionnaire. A validated self-administered FFQ was completed every 4 y beginning in 1991. For this analysis, we used 1991 as the beginning of follow-up with the first administration of the FFQ and concluded follow-up in 2015. We excluded women with a history of cancer, diabetes, myocardial infarction, or stroke, because these diseases may cause weight change and change in dietary intake. In addition, we also excluded those with missing weight status at baseline. Those who did not complete additional questionnaires beyond baseline and those who reported implausible energy intake (<500 or >3500 kcal/d) were also excluded. A total of 68,336 women were included in this analysis and loss to follow-up was ∼10% during the study period. This study was approved by the institutional review boards of Brigham and Women's Hospital and Harvard TH Chan School of Public Health. Completion of the self-administered questionnaire was considered as implied consent.

### Diet assessment

Usual dietary intake was assessed every 4 y using a validated semiquantitative FFQ that included ∼135 items (19). For each food item, a standard portion size was specified with 9 frequency choices from “never or less than once per month” to “≥6 times per day.” The GDQS is a diet quality score comprised solely of food groups that was modified from the Prime Diet Quality Score (20). It was designed to reflect nutrient adequacy and predict major noncommunicable diseases globally (17) (see elsewhere in this Supplement). There are 16 healthy food groups (dark green leafy vegetables, cruciferous vegetables, deep orange vegetables, deep orange fruits, deep orange tubers, citrus fruits, other vegetables, other fruits, legumes, nuts and seeds, poultry and game meat, fish and shellfish, whole grains, liquid oils, low fat dairy, eggs) and 7 unhealthy food groups (white roots and tubers, processed meats, refined grains and baked goods, sugar-sweetened beverages, sweets and ice cream, juices, purchased deep fried foods). Intake was categorized into <1/wk, 1 to <4/wk, and ≥4/wk. Healthy food groups were given points between 0 and 4 for each category of intake depending on the food group. Unhealthy food groups were given 2, 1, and 0 points for the same 3 intake levels with lower intake receiving more points. In addition, the GDQS also includes a red meat group and a full-fat dairy group that are scored differently. Red meat was given 0, 1, and 0 points for intake at the same 3 levels, respectively, and full-fat dairy was given 0, 1, 2, and 0 points for intake of <1/wk, 1 to <4/wk, ≥4/wk to <3/d, and ≥3/d, respectively. This accounts for nutrient contribution with moderate intake but does not reward high intake that may contribute to the risk of chronic diseases. The full GDQS has 25 food groups and a score range of 0–49 points, with a higher score representing a healthier diet. The healthy portion of the GDQS (GDQS+) ranges from 0 to 32 (21) (see elsewhere in this Supplement). For this analysis, we included red meat and full-fat dairy as part of the unhealthy subscore (GDQS−) which has a range of 0–17, with a higher score representing lower intake of unhealthy foods and hence healthier food choices.

In this analysis, the GDQS was compared with 2 other diet quality scores: the Alternate Healthy Eating Index-2010 (AHEI-2010) (22) and the Minimum Dietary Diversity for Women (MDD-W) indicator (20). These were computed for each participant using the same FFQ data. The AHEI-2010 consists of 11 food and nutrient groups. Points were given for higher intakes of healthy groups (vegetables, whole fruits, nuts and legumes, whole grains, polyunsaturated fat, and long-chain n–3 fatty acids) and lower intakes of unhealthy groups (red and processed meats, sugar-sweetened beverages and fruit juice, *trans* fat, and sodium). Points were also given for moderate intake of alcohol. Each component ranges from 0 to 10 points with the total possible score ranging from 0 to 110 points.

The MDD-W has 10 food groups: grains and starchy vegetables, pulses, nuts and seeds, dairy, animal flesh, eggs, dark green leafy vegetables, vitamin A–rich vegetables and fruits, other vegetables, and other fruits. To adapt the original scoring method based on 24-h recall data to our FFQ data, we assigned 1 point for each food group with intake ≥1 serving/d and 0 for less. The MDD-W has a range of 0–10 points (23).

### Outcome assessment

Weight was updated with each biennial questionnaire and self-reported. We calculated 4-y weight change in the same years the FFQ was administered. BMI (in kg/m^2^) was calculated using height collected at baseline and weight reported at each questionnaire cycle. The validity of self-reported weight was assessed in a subsample of women (*n* = 184) via actual weighing 6–12 mo after questionnaire administration. The correlation coefficient between the 2 weights was 0.97 (24).

### Covariates

Age and height were obtained from the baseline questionnaire. Body weight, cigarette smoking (including the number of cigarettes/d), daily number of hours sleeping, weekly number of hours spent on TV watching, menopausal status and postmenopausal hormone use, oral contraceptive use, and pregnancy were self-reported in each biennial questionnaire. Data on leisure-time physical activity were collected every 2 y with 10 questions covering common exercise and recreational activities and their weekly duration. Total physical activity was expressed as metabolic equivalents (METs) per week (25).

### Statistical analysis

The association between 4-y change in GDQS and concurrent 4-y weight change between 1991 and 2015 was examined using multivariable linear models with an unstructured correlation matrix to account for within-person repeated measures. Four-year change in GDQS was categorized into >5 points decrease, >2 to 5 points decrease, ±2 points (considered as no change in score), >2 to 5 points increase, and >5 points increase. Person-years from time periods with missing weight data were excluded. In addition, we censored person time after age 65 y to minimize weight loss and muscle loss associated with aging. Change in GDQS+ was classified into the same categories but change in GDQS− was classified into >2 points decrease, ±2 points (considered as no change in score), and >2 points increase owing to the narrow score range. To minimize the influence of outliers, score changes >99.5% percentile or <0.05% percentile were recoded to values of those specific percentiles.

Participants re-entered the analysis when weight was again reported in subsequent questionnaire cycles. In addition, if pregnancy was reported in a questionnaire period, person-time during that period was excluded.

Multivariable models were adjusted for known confounders for weight change and obesity (26). We included age, menopausal status (pre- or postmenopausal), hormone therapy use (never, past, or current), hours of sleep, BMI at the start of each 4-y interval, and concurrent 4-y changes in lifestyle factors: smoking status (never, former, current: 1–14, 15–24, or ≥25 cigarettes/d), physical activity (METs/wk), hours of sitting per week, and alcohol intake; and BMI and GDQS at the start of each 4-y period. We did not adjust for energy intake because it could be on the causal pathway between GDQS and weight change. We further used Poisson regression to examine 4-y risk of ≥2-kg and ≥5-kg weight gain, and the risk of becoming obese (BMI ≥ 30.0, among nonobese participants), with 4-y change in GDQS, GDQS+, and GDQS−. Separate analyses were conducted for women <50 y old and for women ≥50 y old. Furthermore, we also explored differences in weight change by GDQS comparing those with BMI < 25.0 and those with BMI ≥ 25.0. Interaction with age or BMI was examined by modeling a multiplicative term of GDQS and age or BMI in the model and the likelihood ratio test comparing models with and without the interaction term.

To compare the amount of weight change from changes in GDQS with other diet quality scores, we first standardized 4-y difference in the GDQS, AHEI-2010, and MDD-W and modeled 1 SD in the change of each score. Regression coefficients from the linear model were then compared using the Wald test. All analyses were conducted using SAS version 9.4 (SAS Institute).

## Results

In this cohort of female nurses, the mean ± SD weight gain in all 4-y periods was 1.68 ± 6.26 kg. Women with an increase in GDQS over 4-y periods tended to also increase their physical activity and alcohol consumption, whereas those with a decrease in score also decreased their physical activity level (**Table 1**).

**TABLE 1:** Age-standardized characteristics in the Nurses’ Health Study II population at 1991 baseline and average 4-y change by change of GDQS over the period 1991–2015^1^

		4-y change, points				
	1991 (*n* = 68,336)	<−5 (decrease)	−5 to <−2 (decrease)	−2 to 2 (little change)	>2 to 5 (increase)	>5 (increase)
Age, y	36.3 ± 4.6					
Current weight, kg	66.6 ± 15					
4-y weight change, kg		3.0 ± 7.0	2.4 ± 6.2	1.7 ± 6.0	1.4 ± 6.1	0.7 ± 6.5
BMI, kg/m^2^	24.5 ± 5.3	1.1 ± 2.5	0.9 ± 2.3	0.6 ± 2.2	0.5 ± 2.2	0.3 ± 2.4
Physical activity, METs/wk	20.7 ± 27	−0.7 ± 30.1	−0.4 ± 27.8	−0.5 ± 27.9	0.2 ± 27.8	0.2 ± 27.6
Current smokers	11.9					
Sleep, h/d	5.9 ± 1.0	5.9 ± 1.1	5.9 ± 1.0	5.9 ± 1.0	5.9 ± 1.0	5.9 ± 1.0
TV watching, h/wk	8.9 ± 8.5	0.3 ± 9.0	0.3 ± 8.5	0.2 ± 7.6	0.2 ± 8.5	0.3 ± 8.6
Alcohol, g/d	3.2 ± 6.1	0.4 ± 7.0	0.6 ± 6.9	0.5 ± 6.6	0.8 ± 6.6	0.9 ± 6.7
Total calories, kcal/d	1776 ± 534	−153 ± 509	−77 ± 491	−17 ± 479	73 ± 482	147 ± 505
GDQS	21.6 ± 5.1	−7.4 ± 1.8	−3.4 ± 0.9	0.0 ± 1.2	3.5 ± 0.9	7.8 ± 2.2
GDQS+ (healthy) score	13 ± 4.6	−5.7 ± 2.7	−2.9 ± 2.6	0.0 ± 2.8	2.7 ± 2.5	5.8 ± 2.9
GDQS− (unhealthy) score	8.6 ± 2.5	−1.7 ± 2.4	−0.8 ± 2.3	0.2 ± 2.3	1.0 ± 2.3	2.1 ± 2.5
MDD-W	4.2 ± 1.5	−1.0 ± 1.4	−0.5 ± 1.4	0.0 ± 1.4	0.4 ± 1.4	0.9 ± 1.4
AHEI-2010	48 ± 10.8	−5.5 ± 8.4	−1.7 ± 8.2	2.0 ± 8.2	5.8 ± 8.2	10.4 ± 8.9

^1^Values are means ± SDs for continuous variables and percentages for categorical variables. AHEI-2010, Alternate Healthy Eating Index-2010; GDQS, Global Diet Quality Score; MDD-W, Minimum Dietary Diversity for Women; MET, metabolic equivalent.

After adjusting for potential confounders, women with >5-point increases in GDQS gained less weight (−1.13 kg; 95% CI: −1.19, −1.06 kg) than women with little change in score (**Table 2**). Those with >5-point decreases in GDQS gained 1.03 kg (95% CI: 0.94, 1.11 kg) more than those with little change in score. The association was stronger for women aged <50 y (−1.24 kg; 95% CI: −1.31, −1.16 kg) than for those aged ≥50 y (−0.96 kg; 95% CI: −1.08, −1.84 kg) for >5-point increases (*P*-interaction < 0.05). Among those with a score decrease of >5 points, younger women gained 1.13 kg (95% CI: 1.04, 1.22 kg) compared with 0.81 kg (95% CI: 0.63, 0.98 kg) in older women. The GDQS was more strongly associated with weight change in women with BMI ≥ 25 than with BMI < 25 (*P*-interaction < 0.05 for each age group) (**Supplemental Table 1**). Among women aged <50 y with BMI < 25, each 5-point increase was associated with −0.38 kg (95% CI: −0.41, −0.35 kg) less weight gain. But among younger women with BMI ≥ 25, weight gain was −1.41 kg less (95% CI: −1.47, −1.35 kg) for each 5-point increase in GDQS. Similarly, among women aged ≥50 y, the GDQS had a stronger association with less weight gain among those with higher BMI (−1.00 kg; 95% CI: −1.07, −0.92 kg) than among leaner individuals (−0.31 kg; 95% CI: −0.37, −0.26 kg).

**TABLE 2:** Four-year weight change by different amounts of 4-y change in GDQS in Nurses’ Health Study II participants^1^

	GDQS change, points					
	<−5 (decrease)	−5 to <−2 (decrease)	−2 to 2 (little change)	>2 to 5 (increase)	>5 (increase)	Per 5-point increase
All women						
Age adjusted	0.92 (0.84, 1.00)	0.43 (0.36, 0.49)	Reference	−0.41 (−0.47, −0.35)	−1.06 (−1.13, −1.00)	−0.70 (−0.73, −0.68)
Multivariable^2^	1.03 (0.94, 1.11)	0.47 (0.41, 0.54)	Reference	−0.44 (−0.50, −0.38)	−1.13 (−1.19, −1.06)	−0.77 (−0.80, −0.75)
Women <50 y old						
Age adjusted	1.05 (0.96, 1.14)	0.41 (0.34, 0.48)	Reference	−0.45 (−0.52, −0.38)	−1.16 (−1.24, −1.09)	−0.75 (−0.78, −0.72)
Multivariable^2^	1.13 (1.04, 1.22)	0.45 (0.38, 0.53)	Reference	−0.49 (−0.56, −0.42)	−1.24 (−1.31, −1.16)	−0.83 (−0.86, −0.80)
Women ≥50 y old						
Age adjusted	1.00 (0.83, 1.17)	0.76 (0.63, 0.88)	Reference	−0.05 (−0.16, 0.05)	−0.55 (−0.66, −0.44)	−0.55 (−0.59, −0.50)
Multivariable^2^	0.81 (0.63, 0.98)	0.54 (0.41, 0.67)	Reference	−0.43 (−0.54, −0.31)	−0.96 (−1.08, −0.84)	−0.71 (−0.76, −0.66)

1 Values are weight changes (95% CIs) in kg. GDQS, Global Diet Quality Score.

2 Adjusted for age, time period, change in smoking, oral contraceptive use, menopausal status and postmenopausal hormone use (“all women” analysis only), change in sitting, change in physical activity, change in alcohol intake, baseline GDQS, and sleep duration.

Increase in the GDQS was also associated with a lower risk of gaining 2 kg (**Supplemental Table 2**) or 5 kg in a 4-y period (**Supplemental Table 3**). The RR for 2-kg and 5-kg weight gain for a >5-point decrease in GDQS, compared with little change in score, was slightly but significantly (*P*-interaction < 0.05) stronger for women aged ≥50 y than for those aged <50 y. Specifically, RR for 2-kg gain was 1.18 compared with 1.14 and for 5-kg gain was 1.41 compared with 1.28.

When we explored the healthy (GDQS+) and unhealthy (GDQS−) submetrics of the GDQS, we found that both were associated with weight change (**Supplemental Table 4**). Each 3-point increase in the GDQS+ was associated with −0.19 kg (95% CI: −0.20, −0.17 kg) less weight gain. This association was stronger among women aged <50 y (−0.22 kg; 95% CI: −0.24, −0.20 kg) than among women aged ≥50 y (−0.14 kg; 95% CI: −0.17, −0.11 kg) (*P*-interaction < 0.05). Each 3-point increase was associated with a lower risk of a 2-kg weight gain (RR: 0.97; 95% CI: 0.97, 0.98) and a 5-kg weight gain (RR: 0.95; 95% CI: 0.94, 0.96), with no significant difference between younger and older women (**Supplemental Table 5**).

On the other hand, a >2-point decrease in GDQS− (representing increased intake of unhealthy foods), compared with little change, was associated with 1.23 kg more weight gain (95% CI: 1.16, 1.29 kg) (Supplemental Table 4). The association was stronger, however, for women aged ≥50 y (1.33 kg; 95% CI: 1.21, 1.46 kg) than for women aged <50 y (1.19 kg; 95% CI: 1.12, 1.27 kg) (*P*-interaction < 0.05). The association of a >2-point decrease in GDQS− and risk of weight gain was also stronger in older than in younger women for 2 kg and 5 kg weight gain (*P*-interaction < 0.05 for both): for 2 kg, RR was 1.20 compared with 1.13; and for 5 kg, RR was 1.43 compared with 1.24 (Supplemental Table 5).

Increase in the GDQS was associated with a lower risk of developing obesity in a 4-y period (**Table 3**). The RR for each 5-point increase was 0.84 (95% CI: 0.82, 0.95). No significant interaction was observed with age. Similarly, the GDQS+ was associated with a lower risk of obesity (RR for 3 points increase: 0.95; 95% CI: 0.94, 0.96), with no apparent difference by age (Supplemental Table 5). However, the GDQS− had a stronger association with the risk of obesity among older women (RR for >2 points decrease: 1.53; 95% CI: 1.42, 1.66) than among younger women (RR for >2 points decrease: 1.29; 95% CI: 1.25, 1.35) (*P*-interaction < 0.05).

**TABLE 3:** RRs (95% CIs) of developing obesity in 4 y by different amounts of 4-y change in GDQS in Nurses’ Health Study II participants^1^

	GDQS change, points					
	<−5 (decrease)	−5 to <−2 (decrease)	−2 to 2 (little change)	>2 to 5 (increase)	>5 (increase)	Per 5-point increase
All women						
Age adjusted	1.40 (1.34, 1.46)	1.13 (1.09, 1.18)	1.00 (reference)	0.93 (0.90, 0.97)	0.84 (0.81, 0.88)	0.85 (0.84, 0.86)
Multivariable^2^	1.32 (1.26, 1.37)	1.13 (1.08, 1.17)	1.00 (reference)	0.89 (0.86, 0.93)	0.77 (0.74, 0.81)	0.84 (0.82, 0.85)
Women <50 y old						
Age adjusted	1.43 (1.36, 1.51)	1.10 (1.05, 1.16)	1.00 (reference)	0.89 (0.85, 0.94)	0.79 (0.75, 0.83)	0.82 (0.81, 0.84)
Multivariable^2^	1.31 (1.25, 1.37)	1.08 (1.03, 1.13)	1.00 (reference)	0.88 (0.84, 0.92)	0.76 (0.72, 0.80)	0.83 (0.82, 0.85)
Women ≥50 y old						
Age adjusted	1.47 (1.30, 1.66)	1.28 (1.16, 1.41)	1.00 (reference)	0.99 (0.90, 1.08)	0.91 (0.83, 1.00)	0.85 (0.82, 0.89)
Multivariable^2^	1.38 (1.23, 1.55)	1.26 (1.15, 1.38)	1.00 (reference)	0.93 (0.85, 1.01)	0.80 (0.73, 0.88)	0.83 (0.80, 0.86)

1 GDQS, Global Diet Quality Score.

2 Adjusted for age, time period, change in smoking, oral contraceptive use, menopausal status and postmenopausal hormone use (“all women” analysis only), change in sitting, change in physical activity, change in alcohol intake, baseline GDQS, and sleep duration.

**FIGURE 1:**
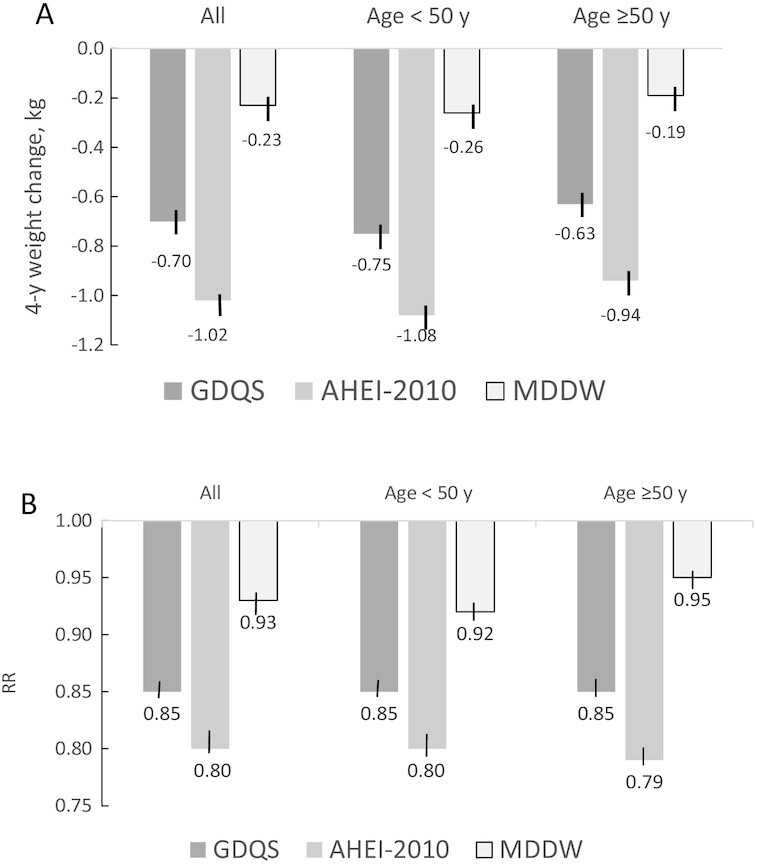
Multivariable-adjusted 4-y weight change (A) or 4-y risk of obesity (B) by concurrent 1-SD increase in diet quality scores (adjusted for the same variables as in Table 2) in Nurses’ Health Study II participants. (A) Weight change (kg) (*P* values comparing GDQS with AHEI-2010 or with MDD-W were <0.05). (B) RR for obesity (*P* values comparing GDQS with AHEI-2010 or with MDD-W were <0.05). AHEI-2010, Alternate Healthy Eating Index-2010; GDQS, Global Diet Quality Score; MDD-W, Minimum Dietary Diversity for Women.

When we compared the GDQS with the AHEI-2010 and MDD-W, we observed significant associations between all 3 diet quality scores and weight change (**Figure 1**A), risks of 2-kg and 5-kg weight gain (**Supplemental Table 6**), and risk of obesity (Figure 1B). In pairwise comparisons, all associations were significantly stronger for AHEI-2010 than GDQS, and stronger for GDQS than MDD-W (all *P* values < 0.05). However, the difference between the AHEI-2010 and GDQS for RR for obesity was small (0.80 compared with 0.85 for all women), and the difference for weight change was 0.32 kg among all women.

## Discussion

In this analysis of US women, increase in the GDQS over 4-y periods was associated with less concurrent weight gain and lower risk of obesity. The association for weight change was stronger among women aged <50 y, but there was no age difference for the risk of obesity.

A number of prospective observational studies from Europe (9, 27), Australia (10, 28), and the United States (29), including African-American women (30), have found inverse association between healthy diets and weight change or risk of overweight and obesity. Adherence was commonly measured with diet quality indexes (10, 28, 29) or from derived dietary patterns (30) and included various Mediterranean diet scores (9, 27). Although the characteristics of the metrics vary, they generally emphasized higher intakes of fruits, vegetables, whole grains, and fish and lower intakes of refined grains, red and processed meats, and added sugar. In addition, a randomized trial among reproductive-age women in Australia showed an improvement in diet quality was associated with less weight gain (12). These results suggest that dietary characteristics that favor weight management can have a fair amount of variation within some general principles.

A number of mechanisms may explain the association between healthy diets and weight change. Diets high in fruits and vegetables tend to be lower in energy density relative to their volume and therefore promote satiety and hence less energy intake per meal (31). Moreover, the lower glycemic load of minimally processed carbohydrates does not produce large postprandial fluctuations in insulin concentrations and thus could sustain satiation (13). A plant-rich diet contains more fermentable fibers and leads to higher concentrations of absorbable SCFAs produced by gut microbes (32). These SCFAs are capable of crossing the blood–brain barrier and regulate appetite (33). Therefore, healthy dietary patterns with their focus on minimally processed plant foods may modulate weight change trajectories.

This analysis has several strengths. Examining weight change in 4-y periods avoided short-term weight fluctuations caused by diet changes that were not sustained. The large sample size allowed for adequate power to examine both women of reproductive age (<50 y) and older women (≥50 y). We had repeated data on numerous potential confounders to minimize confounding. However, because data were obtained from self-report, some amount of measurement error was unavoidable. Because members of the NHS II were nurses, their knowledge and awareness of their health-related behaviors would likely result in less reporting error than among the general public.

The GDQS was constructed to capture dietary characteristics that would predict both nutrient adequacy and risk of chronic disease–related outcomes. Therefore, it would be expected to have a stronger association with weight change than the MDD-W, which was constructed to reflect only micronutrient adequacy in low-income countries. On the other hand, other metrics could capture dietary characteristics that are more strongly associated with weight change and could perform better than the GDQS. As observed in this analysis, a 1-SD increase in the AHEI-2010 was more strongly associated with weight change than a 1-SD increase in the GDQS. Nevertheless, our analysis showed that an achievable amount of improvement in the GDQS was associated with significantly less weight gain of a meaningful magnitude. In addition, unlike the AHEI-2010, the GDQS does not require sophisticated dietary collection and analysis technology, and hence can be used in a wider range of settings. Also, the GDQS is a metric that is useful in the global context and has the advantage of comparability across global studies. Therefore, results from this analysis adequately support using the GDQS as a metric to gauge diet quality for the purpose of weight management. Although environmental sustainability was not a primary focus in constructing the GDQS, its emphasis on plant foods and minimally processed foods does encompass sustainable eating habits to a considerable extent.

In conclusion, improvement in diet quality as measured by the GDQS was associated with less weight gain in US women, with the association stronger for those of reproductive age. An inverse association was also observed between the GDQS and risk of obesity with similar magnitude across age groups.

## Notes

Funding for the research was provided by FHI Solutions, recipient of a Bill & Melinda Gates Foundation grant to support Intake—Center for Dietary Assessment and by NIH grant U01 CA176726.

Author disclosures: TTF is an Associate Editor for the Journal of Nutrition and played no role in the Journal’s evaluation of the manuscript. All other authors report no conflicts of interest.

Published in a supplement to *The Journal of Nutrition*. Publication costs for this supplement were funded by the Bill & Melinda Gates Foundation in conjunction with FHI Solutions, recipient of a Bill & Melinda Gates Foundation grant to support Intake—Center for Dietary Assessment. The opinions expressed in this publication are those of the authors and are not attributable to the sponsors or the publisher, Editor, or Editorial Board of *The Journal of Nutrition*. The Supplement Coordinators for the supplement publication were Megan Deitchler, Intake—Center for Dietary Assessment at FHI Solutions, Washington, DC; and Sabri Bromage, Harvard TH Chan School of Public Health, Boston, MA.

The GDQS research initiative was launched by Intake – Center for Dietary Assessment. The research was led by Harvard T.H. Chan School of Public Health, Department of Nutrition and carried out in collaboration with researchers at the National Public Health Institute (INSP), Mexico. Funding for the research was provided by FHI Solutions, recipient of a Bill & Melinda Gates Foundation grant to support Intake – Center for Dietary Assessment.

Abbreviations used: AHEI-2010, Alternate Healthy Eating Index-2010; GDQS, Global Diet Quality Score; MDD-W, Minimum Dietary Diversity for Women; MET, metabolic equivalent; NHS, Nurses’ Health Study.
